# Residential summer camp: a new venue for nutrition education and physical activity promotion

**DOI:** 10.1186/1479-5868-10-64

**Published:** 2013-05-24

**Authors:** Alison K Ventura, Barry A Garst

**Affiliations:** 1 Department of Nutrition Sciences, College of Nursing and Health Professions, Drexel University, Philadelphia, PA, USA; 2 American Camp Association, Martinsville, IN, USA

**Keywords:** Healthy eating, Physical activity, Children, Residential summer camps, Obesity prevention

## Abstract

**Background:**

Millions of children attend residential summer camps each year. However, few studies have examined the potential of camps for obesity prevention efforts. Research in the domain of positive youth development has shown that camp programs as short as one week have both short- and long-term positive effects on self-esteem, self-efficacy and other youth outcomes. The objective of the present study was to highlight the potential of resident camps as promising venues for the promotion of healthy eating and physical activity behaviors in the children who attend.

**Methods:**

Data for this study came from the American Camp Association 2007 Emerging Issues Survey. This survey assessed camp professionals’ perspectives on a diverse array of issues, including the healthy eating and physical activity of children. Data analysis focused on responses from 247 camp professionals whose camps offered resident camp programs.

**Results:**

Descriptive and Chi-square statistics were calculated. Ninety-two percent of camp professionals reported that the healthy eating and physical activity of campers was an “important” or “very important” issue for camps. The majority of camps reported offering vegetarian options, healthy snacks and salad bars, and allergen-free options. Additionally, 86% of camp professionals indicated that they had implemented one or more strategies to address concerns related to the unhealthy eating behaviors of children, with top strategies including increasing the availability of fruits and vegetables, increasing the availability of healthy drink options, and improving the nutritional quality of menus. Fewer camp professionals (50%) indicated they had implemented strategies to increase children’s physical activity levels, but many professionals indicated that their camp programs were inherently active and additional strategies to promote physical activity were not necessary. Associations were found between camp affiliation and food options available to campers.

**Conclusions:**

The majority of camp professionals believe the healthy eating and physical activity of children are important issues for camps and have implemented strategies to address these issues. An important question for future research is to examine whether these strategies are effective in promoting healthy eating and physical activity behaviors in children, as well as ways that camp programs could be improved.

## Background

The diets and physical activity behaviors of American children and adolescents need improvement. Eighty-six percent of young children consume a sweetened beverage or dessert daily [1] and over 80% of 4-18-year-old children do not meet recommendations for fruit or vegetable intakes [2]. Additionally, 8-18-year-old children watch an average of 4.5 hours of television daily [3] and over half of adolescents do not meet physical activity recommendations [4]. The negative impact of these concerning trends is illustrated, in part, by the increasing prevalence of obesity among children and adolescents [5].

Interventions aimed at improving children’s eating and physical activity behaviors have focused on a number of contexts within which youth spend their time, including homes [6], schools [7], and community-based organizations [8]. Less attention has been paid to the potential of summer camps, despite the fact that, as an institution, summer camps rank second only to schools in the number of children who attend [9]. The American Camp Association (ACA) serves as a knowledge center for the camp community and offers the only recognized accreditation program for U.S. camps that meet up to 300 standards for health, safety, and program quality. Camps accredited by the ACA serve a diverse array of children: 90% of these camps offer financial assistance to children who are from economically deprived families or experience other hardships that may interfere with their ability to attend camp [10]. Additionally, 5% of ACA-Accredited camps specifically target youth who are at-risk, 42% target youth from economically-deprived families, and 60% target children with special medical needs [10]. Thus, summer camps provide access to a large number of children, many of whom are at higher risk for poor dietary intake and high prevalence of obesity.

The majority of summer camps in the U.S. are residential summer camps. Resident camps generally have the following characteristics: 1) sessions vary in length, 2) the program is operated and staffed by the camp, 3) supervision of individual campers is a camp responsibility, 4) campers stay overnight, and 5) camp is responsible for campers 24 hours a day [11]. Several common elements of resident camp programs make them viable settings for interventions to improve children’s eating and physical activity behaviors. First, resident camps are a controlled environment where camp administrators determine the foods offered to campers at meals and the activities in which campers participate. Second, a hallmark feature of resident camps is the provision of developmentally appropriate supports and opportunities aimed at fostering skill building and personal growth for children [12]. For many children, camp provides supportive peer and adult relationships and novel resources and experiences not available in their home environments. Third, camps are sustained and immersive experiences that have the intensity and duration to have a substantial impact on children’s developmental outcomes [13].

A strong body of research suggests that camp experiences as short as one week [13] can increase children’s locus of control and general self-efficacy, and positive effects of camp experiences on children’s psychosocial development are maintained for months [14] and years [15] beyond the camp experience. One of the first large-scale efforts to document the psychosocial outcomes of the camp experience was the ACA’s National Youth Development Outcomes study, in which over 5,000 campers, staff, and parents from a representative national sample of camps were asked about the ways in which campers benefitted from their camp experience [14]. Campers, parents, and camp staff all reported significant increases in campers’ self-esteem, peer relationships, independence, exploration, leadership, friendship skills, values and decisions, social comfort, and spirituality between the pre- to immediate post-camp assessments. Additionally, campers and parents reported maintenance of, or in some cases further increases in, these gains 6 months after camp. That these changes were consistent across camper, parent and camp staff reports strongly suggests the camp experience was the source of sustainability in these outcomes, and reinforce the potential of camps for positive youth development [16].

The ability of residential summer camps to promote healthy eating and physical activity behaviors in children has received a paucity of research attention. While a very small percentage of camps (1%) are weight loss camps that specifically target obese children [17,18] most camps either focus on a specific activity or offer a broad array of activities that include aquatics, arts, and crafts, sports, environmental exploration, outdoor adventure, and academics. Whether and how these camps make an intentional effort to promote healthy eating and physical activity behaviors in their campers has not been empirically studied.

As a first step towards understanding how the residential summer camp experience may influence children’s eating and physical activity behaviors, the present study explored camp professionals’ perceptions of: 1) the importance of promoting healthy eating and physical activity behaviors in children; 2) the food options available at their camps; and 3) any strategies they have implemented at their camps to promote healthy eating behaviors and physical activity in their campers. This study aimed to provide a foundation for future research focused on understanding how the residential summer camp experience can be optimized to have a positive impact on the healthy eating and physical activity behaviors of children.

## Methods

### Participants

Data for this study came from the ACA 2007 Emerging Issues Survey. In November 2007, camp professionals (owners, directors, administrators; *N* = 2,353) who were ACA members were invited to participate in an online survey^a^. A total of 367 surveys were completed (response rate = 16%).

### Survey design

Survey questions were developed by the ACA in consultation with research experts from the ACA’s Committee for the Advancement of Research and Education, which is composed primarily of university faculty from a variety of youth-related disciplines. The survey was designed to assess camp professionals’ perceptions of emerging camp issues and trends. Survey questions focused on 12 issue areas: 1) Security; 2) Parent Communication; 3) Medical Insurance and Medication; 4) Healthy Eating and Physical Activity of Children; 5) Electronics and Social Networks; 6) Severe Weather; 7) Programming; 8) Problem Behaviors; 9) Staff Recruitment, Screening, and Training; 10) Crisis Management; 11) Outcome Evaluation; and 12) Camper Pregnancies. Camp professionals also provided data on the following demographic variables: programs offered (residential, day camp, or family programs), camp affiliation (independent for-profit, independent not-for-profit, agency, or religious), current position at camp, number of years experience in this position, gender, age group, and ethnic heritage. As a secondary analysis of de-identified data, this study was exempt from Institutional Review Board approval.

With respect to the Healthy Eating and Physical Activity of Children issue area, camp professionals were asked three questions:

1) Which of the following food options do you provide to campers?

2) In the past three years, how has your camp addressed concerns related to unhealthy eating behaviors of children?

3) In the past three years, what strategies have you tried to increase children's physical activity level at camp?

Camp professionals were provided with a variety of response options, but also allowed to select “Other” and provide a qualitative response to each question posed. Tables 1, 2 and 3 list the possible response options for Questions 1, 2 and 3, respectively. Definitions were not provided for any of the terms used (for example, “healthy eating” or “healthy snacks”).

**Table 1:** **Food options offered by camps with residential summer camp programs**^**1 **^**(*n*** **= 247)**

	**Percent**	***n***
Vegetarian	89.1	220
Healthy snacks	76.5	189
Salad bar	70.9	175
Other allergy-free options	55.5	137
Peanut-free / Soy Nut Butter	42.1	104
Vegan	25.1	62
Pork free	22.3	55
Portion controlled	19.8	49
Organic/Local	13.8	34
Kosher	13.0	32
Calorie controlled	10.1	25
Other	14.2	35
I do not offer any of the options listed above	2.0	5

^1^ Respondents could select more than one option.

**Table 2:** **Strategies used by camps with residential summer camp programs to address unhealthy eating behaviors of children**^**1 **^**(*n*** **= 247)**

	**Percent**	***n***
Increased fruits and vegetables	73.3	181
Increased healthy drink options (milk, water)	55.1	136
Re-designed daily menus to incorporate healthier foods	55.1	136
Reduced sweets/sugary foods	45.7	113
Reduced fried foods	43.3	107
Provided more healthy food options in canteen	36.4	90
Reduced candy, soda, and other “junk food” in canteen	33.2	82
Prohibited care packages containing food	27.1	67
Offer low/no fat alternatives where possible	16.6	41
Served smaller meal portions	6.9	17
Encouraged small-portion meals and/or healthy snacks	3.6	9
Other	13.0	32
No new strategies have been used	13.8	34

^1^ Respondents could select more than one option.

**Table 3:** **Ways in which camps have tried to increase children's physical activity level at camp **^**1 **^**(*n*** **= 247)**

	**Percent**	***n***
Encouraged staff to be more physically active in order to set a better example for the campers	39.3	97
Increased the frequency of program options that incorporate vigorous physical activity	26.7	66
Provided coaching/instruction to campers on the value of physical activity	16.6	41
Required participation in programs that incorporate vigorous physical activity	14.2	35
Decreased the frequency of any minimally physically active program options	4.9	12
Other	6.1	15
No new strategies have been used	50.0	123

^1^ Respondents could select more than one option.

### Statistical analysis

For the present study, only data from camp professionals whose organizations offered a residential summer camp program were analyzed (*n* = 247). Additionally, data analysis focused on survey items that pertained to the healthy eating and physical activity of children and the demographic characteristics of respondents. Quantitative data were analyzed using SPSS version 20 (Chicago, IL). Descriptive statistics were calculated to summarize participant responses. Chi-square tests were used to examine the association between camp affiliation and healthy eating and physical activity offerings at camps. A significance level of *p* < .05 was used for all 2-sided tests of no association. Qualitative data from open-ended questions were sorted into thematic categories using constant comparison within the framework of grounded theory [19].

## Results

### Sample characteristics

Camp professionals reported that their camps served an average of 1,060 ± 1,658 (range = 61 to 21,940) children annually. Combined, the camps participating in this survey served a total of 259,598 children per year. Table 4 presents demographic information for the sample. Equal proportions of male and female camp professionals participated in this survey. Thirty percent of camp professionals were between 45 and 54 years of age and 95% were white. Approximately two-thirds (62.3%) of respondents were camp directors. Thirty-nine percent of camps were affiliated with an agency, (e.g., YMCA or Boy/Girl Scouts), while the remaining 61% were dispersed among independent-for-profit (18.2%), independent not-for-profit (22.7%), or religious (19.4%) affiliations. While all camps included in this study offered a residential program, many also offered day camps, trip or travel camps, and family camps.

**Table 4:** **Characteristics of camp professionals participating in the 2007 American Camp Association Emerging Issues Survey (*n*** **= 247)**

	**Percent**	***n***
Gender		
Male	49.4	122
Female	47.8	118
No Response	2.8	7
Age group		
18-24 years	1.2	3
25-34 years	23.5	58
35-44 years	27.9	69
45-54 years	30.0	74
55-64 years	15.0	37
>65 years	1.2	3
No response	1.2	3
Race/Ethnicity		
Black	0.4	1
White	94.7	234
Other (e.g. multiracial)	4.9	12
Years of experience in current camp position		
< 2 years	11.7	29
2 to 5 years	25.9	64
6 to 10 years	24.3	60
>10 years	38.1	94
Position at Camp		
Camper owner or operator	11.3	28
Camp director	62.3	154
Agency executive	15.4	38
Camp administrative staff ^1^	9.3	23
Other	1.6	4
Camp Affiliation		
Agency (e.g. YMCA, Girl or Boy Scouts, Camp Fire)	38.9	96
Independent for-profit	18.2	45
Independent not-for-profit	22.7	56
Municipality or government	0.8	2
Religiously affiliated	19.4	48
Type of Program Offered^2^		
Residential camp	100.0	247
Day camp	30.8	76
Trip/travel camp	32.0	79
Outdoor/environmental education center	29.1	72
Conference/retreat center	37.2	92
Day use programs	27.5	68
Family camp	36.8	91

^1^ Assistant director, program director, business manager, etc.

^2^ Respondents could select more than one option.

### Perceptions of the importance of healthy eating and physical activity

Camp professionals were asked to rate the importance of the healthy eating and physical activity of campers based on a 4-point Likert scale (1 = very unimportant; 2 = unimportant; 3 = important; 4 = very important). Forty-one percent (*n* = 102) of camp professionals reported that the healthy eating and physical activity of campers was “very important,” whereas 51% (*n* =126) of respondents said that healthy eating and physical activity of children was “important” and 8% (*n* =19) reported that this issue was “unimportant” or “very unimportant.” Camp professionals’ ratings of the importance of healthy eating and physical activity of children were not associated with camp affiliation.

### Food options at camps

Table 1 presents camp professionals’ responses to the question: “Which of the following food options do you provide to campers?” Camp professionals were provided with a list possible options, of which more than one could be selected. The majority of camp professionals reported offering vegetarian options, healthy snacks, salad bars, and allergen-free options. Camp professionals who indicated “Other” (14.2%, *n* =35) provided additional qualitative information about their food options. The majority of these responses centered around three themes: 1) providing options for celiac disease, lactose intolerance, diabetes and other medical conditions (*n* = 30); 2) providing low-salt or -sugar meal options (*n* = 9); and 3) willingness to tailor food options to campers’ individual needs (*n* = 13).

A significantly higher percentage (26.7%, *n* = 12) of professionals from independent for-profit camps reported that their camp offered local/organic food options compared to professionals from agency camps (8.3%, *n* = 8; *χ*^2^ = 8.7, *p* = .033). Significantly greater proportions of professionals from independent not-for-profit camps reported that their camp offered calorie-controlled meals (23.2%, *n* = 13) compared to professionals from agency camps (5.2%, *n* = 5; *χ*^2^ = 13.8, *p* = .003). A significantly greater proportion of professionals from independent not-for-profit camps also reported that their camp offered portion-controlled meals (35.7%, *n* = 20) compared to professionals from agency (12.5%, *n* = 12) and independent for-profit camps (8.9%, *n* = 4; *χ*^2^ = 17.0, *p* < .001).

### Strategies to address unhealthy eating behaviors of children

Table 2 presents camp professionals’ responses to the question: “In the past three years, how has your camp addressed concerns related to the unhealthy eating behaviors of children?” Camp professionals were provided with a list possible options, of which more than one could be selected. Approximately 86% of camp professionals indicated that they had implemented one or more strategies to address concerns related to unhealthy eating behaviors of children. Top strategies included increasing the availability of fruits and vegetables (73.3%, *n* = 181), increasing the availability of healthy drink options (55.1%, *n* = 136), and redesigning daily menus to incorporate healthier foods (55.1%, *n* = 136).

Camp professionals who indicated “Other” (13.0%, *n* = 32) provided additional qualitative information. Responses centered around four themes: 1) specific strategies focused on the quality and type of food served, including increased purchase of local or organic foods and increased availability of whole grains (*n* = 16); 2) food options have always been healthy, thus new strategies were not needed (*n* = 12); 3) prohibition of certain foods in care packages or canteens/camp stores (*n* = 10); and 4) adoption of nutrition education programing (*n* = 8). Responses related to increased nutrition education included offering both formal and non-formal education in the form of nutrition workshops and electives or encouraging counselors to model healthy eating habits.

A significantly higher percentage (31.1%, *n* = 14) of professionals from independent for-profit camps reported that their camp “offered low/no fat alternatives where possible” compared to professionals from religiously affiliated camps (8.3%, *n* = 4; *χ*^2^ = 9.9, *p* = .020). No other associations between affiliation and strategies to address unhealthy eating behaviors of children were found.

### Promotion of physical activity at camps

Table 3 presents camp professionals’ responses to the question: “In the past three years, what strategies have you tried to increase children's physical activity level at camp?” Camp professionals were provided with a list possible options, of which more than one could be selected. Approximately fifty percent (*n* = 123) of camp professionals indicated that they had implemented one or more strategies to increase children’s physical activity levels. The strategy with the highest endorsement (39.3%, *n* = 97) was “encouraging staff to be more physically active in order to set a better example for the campers.” Eight percent (*n* = 20) of camp professionals noted that their camp program has always included many opportunities for physical activity, with several also indicating that they did not feel strategies for further increasing children’s levels of physical activity were necessary.

A significantly higher percentage (23.2%, *n* = 13) of professionals from independent not-for-profit camps reported that their camp “required children participate in programs that incorporate physical activity” compared to professionals from agency camps (6.2%, *n* = 5; *χ*^2^ = 9.4, *p* = .025). No other associations between affiliation and strategies to increase physical activity of children were found.

## Discussion

Residential summer camps serve millions of children each year [10], and have been shown to have a positive impact on children’s development [16]. Previous research suggests the summer months may be a time when children are more vulnerable to inactivity [20,21] and unhealthy eating behaviors [21]. Children also tend to have higher gains in BMI during the summer months compared to the school year [22]. We propose that resident camps have the potential to reverse these trends given their duration, intensity, and provision of developmentally appropriate supports and opportunities. However, more research is needed to understand the effects resident camps currently have on children’s health behaviors, and the ways in which camp programs can be improved to have positive short- and long-term effects on children’s eating, physical activity, and weight status.

The present study illustrated that the majority of camp professionals believe the healthy eating behaviors and physical activity of children is an important issue for camps. These findings are promising because they show that camp professionals value the promotion of healthy eating behaviors and physical activity in their campers and are taking measures to actively encourage these behaviors at camp. Research on factors that contribute to children’s health outcomes in other settings suggests that the strategies camp professionals reported would be effective in promoting healthy behaviors. For example, availability of fruits and vegetables is one of the strongest predictors for children’s fruit and vegetable intakes in both home- and school-based settings [23-25] and repeated exposure to healthy foods promotes acceptance [26-28]. Additionally, availability of physical activity opportunities, parents’ logistic support for physical activity, and parent modeling of physically active behaviors are all associated with higher levels of physical activity in chidren [29-32]. Thus, the findings that over 70% of camp professionals reported increasing the availability of fruits and vegetables and over 25% reported increasing camp staff modeling of physical activity and frequency of physically active programing over the past three years suggests that camps are providing children with opportunities to consume healthful foods and participate in physical activity in a supportive and encouraging environment.

Previous research suggests that many of the food options that camp professionals indicated were available at their camps would also promote healthful dietary patterns for campers. For example, almost 90% of camps offer vegetarian meals and 25% offer vegan meals, both of which have been shown to promote lower intakes of cholesterol, saturated fat and total fat, and higher intakes of vegetables, fruits and fiber for children and adolescents [33,34]. Additionally, the majority of camps reported offering healthy snacks, which may contribute positively to intake of key nutrients and lower risk for obesity [35]. Other healthy options that received far fewer endorsements were organic/local foods and portion/calorie controlled meals. Both would be beneficial to promote in camp settings given evidence for health benefits of organic foods [36], and the potential of portion/calorie controlled meals for promotion of age-appropriate energy intake [37,38].

A limitation of these data is that they represent camp professionals’ perceptions. Camp professionals reported implementing a number of strategies to promote healthy eating and physical activity in children, but the fidelity to which these strategies were implemented and the quality of these changes is unknown. For example, we do not have data to determine whether camp professionals that reported redesigning their menus did so under the guidance of a registered dietician or whether the healthy eating and physical activity opportunities reported by these camp professionals were effective in influencing behavior within a camp setting. Limited research in other samples suggests that camp is effective in promoting physical activity in the short-term. Children attending both day and resident camps meet or exceed physical activity guidelines recommended by the United States Department of Health and Human Services [39,40], and children attending summer camps are more physically active than children who do not attend camps [21]. However, further research is needed to evaluate what children are eating and how much physical activity children are participating in at camps, as well as whether these behaviors are sustained or changed – for better or worse – after the camp experience.

Camp affiliation was associated with camp professionals’ reports of food options available to campers. Specifically, a greater number of independent for-profit camps offered local/organic food compared to agency camps and added low/no fat alternatives compared to religiously affiliated camps. Additionally, a greater number of independent not-for-profit camps endorsed restrictive food options including calorie- and portion-controlled meals compared to agency and independent for-profit camps.

These associations may reflect differences in the resources available to camps of differing affiliations. Independent for-profit camps typically charge higher tuition than other types of camps, thus can afford a higher level of expenses. In 2008, ACA-Accredited independent for-profit camps reported an average of $153,790 in food and related expenditures, whereas agency, independent not-for-profit, and religious camps reported approximately $109,900, $95,445, and $74,664 in food and related expenses, respectively [41]. Thus, independent for-profit camps have higher food budgets that may allow for local/organic foods, low/no fat alternatives and other healthier, but more expensive food options. Additionally, in 2009, independent for-profit camps reported paying a significantly higher weekly wage to their Food Service Director/Supervisor compared to camps of other affiliations [42], which may suggest that the individuals in charge of food-related decisions at independent for-profit camps have more education, training, and experience, making them better equipped to plan healthy menus.

Differences in resource availability are especially concerning when characteristics of the youth/families served by camps of differing affiliations are taken into consideration. Eighty-nine percent of children attending independent for-profit camps are from high income families [41], thus these families may already have the resources and education to provide their children with healthy diets and opportunities to be physically active. In contrast, 80% and 67% of agency and independent not-for-profit camps serve youth from low-income families, and 41 and 44% serve youth from families in poverty (compared to less than 10% of independent for-profit camps) [41]. Thus, agency and independent not-for-profit camps are serving youth at higher risk for poor nutrition, inactivity and obesity, but these types of camps may have fewer resources available to promote healthy eating and physical activity opportunities. These camps may benefit from greater access to resources that allow them to provide the same diet quality and physical activity opportunities as camps that serve higher income families. These camps may also need strategies for securing healthy food resources. These strategies could include guidance related to how to ask donors for healthy options or how maximize the healthfulness of their menu within the constraints of their food budgets or food donations.

A recent statement from the American Academy of Pediatrics (AAP) Council on School Health provided recommendations for creating healthy camp experiences [43]. Within this statement, the AAP stressed that camp communities should promote healthy lifestyles for children by: 1) offering foods that follow federal guidelines for school nutrition, 2) encouraging staff to model healthful behaviors for campers, 3) limiting food used as reward, 4) ensuring children obtain at least 30 minutes of physical activity daily, and 5) offering water instead of sugar-sweetened beverages, including sports drinks [43]. Although the present study suggests that many camps are already following some or all of these recommendations, policies do not currently exist to ensure that all camps embrace these practices. Findings from this and future studies may serve as an important basis for the development of such policies. Policy-level changes could also aim to reduce disparity between offerings at for-profit and not-for-profit or agency camps by providing additional subsidies or supports to camps that serve children at higher risk for obesity. Camps are currently eligbile to participate in the USDA’s Summer Food Service Program [44], but participation rates are low [45]; future research supporting the need for improved nutrition options at camps that reach underserved populations may help increase awareness and participation in such programs and could also help guide adaptations to the USDA’s Summer Food Service Program to ensure that it fits camps’ needs. Camps are promising venues for the promotion of healthy eating and physical activity behaviors, and future research will serve to elucidate the particular ways in which this promotion should occur to optimize health outcomes for the children who attend.

These data are not without limitations. As noted above, these data are self-reported and reflect the perceptions of camp professionals. Another limitation of these data is the low response rate (16%), which is comparable to what is often seen with web-based survey designs [46,47], but may limit the generalizability of our findings. Unfortunately, we do not have demographic information for non-respondents, thus cannot compare respondents to non-respondents to assess whether our sample was biased. Further research is needed to understand whether our findings are representative of the greater population of camp professionals and camps.

## Conclusions

Residential summer camps reach a large number of youth at at time when they are susceptible to inactivity, poor diet quality, and accelerated weight gain. The idea that structured summer programs such as resident camps can promote healthy eating and physical activity behaviors in children is not new [48], but has received little attention when compared to research focused on other youth-serving organizations.

We propose that resident camps may be particularly effective sites for obesity prevention efforts because camp programs are relatively short-term compared to school-based interventions, but have been shown to have the duration, intensity and positive supports to instill meaningful and lasting impact on children’s development. Additionally, camp staff may be particularly influential models for campers’ eating and physical activity behaviors because camp staff are perceived by children to be both a peer and an authority figure, creating a relationship unique to that of other caregivers [16]. Thus, children may be especially receptive to camp programs and camp staffs’ behaviors in ways that are distinct from other youth serving organizations.

Residential camps have been shown to be effective sites for prevention within other domains of youth development including reduction of anxiety [49] and reduction of camper injuries and illness [50]. Additionally, policy-level changes related to implementation of structured summer programs have been effectively used to counter issues such as summer learning decline [51]. We believe camp programs can be used in a similar manner to promote healthy eating and physical activity behaviors and reduce children’s risk for obesity, but whether the unique environment of camps can be effectively used in this capacity is an important question for future research. The present study illustrated that camps are interested and invested in this issue. Further research is needed to fully understand the current impact camps have on children’s health behaviors and the ways in which camp programs and policies related to camp programs can be futher improved to promote health and prevent obesity.

## Endnote

^a^Survey results can be accessed at: http://www.surveymonkey.com/sr.aspx?sm=GZn6UTvY94DT78dVlyXgQ7Lefq78E_2b4jL6SBgIRmomg_3d.

## Competing interests

Both authors declare that they have no competing interests.

## Authors’ contributions

BAG was involved in the acquisition of the data. AKV analyzed and interpreted the data. Both authors drafted the manuscript and critically revised it for important intellectual content. Both authors read and approved the final manuscript.
